# Review: developments in the creep of materials over a period of more than a century

**DOI:** 10.1007/s10853-025-10922-6

**Published:** 2025-05-22

**Authors:** Terence G. Langdon

**Affiliations:** 1 Materials Research Group, Department of Mechanical Engineering, University of Southampton, Southampton, SO17 1BJ UK; 2 Departments of Aerospace and Mechanical Engineering and Materials Science, University of Southern California, Los Angeles, CA 90089‑1453 USA

## Abstract

Studies of creep may be traced back for more than 100 years and this extensive experimentation has produced a comprehensive understanding of the various flow mechanisms occurring in the steady-state or secondary stage of creep. These mechanisms range from diffusional creep and Harper-Dorn creep at low stresses to dislocation processes such as glide and climb at higher stresses and to grain boundary sliding where the rate is dependent primarily upon the grain size. This review examines the nature and characteristics of these flow processes and then demonstrates that the theoretical predictions are generally in good agreement with the experimental data. Finally, two examples are presented, in the fields of structural engineering and glaciology, to illustrate the potential for making significant new contributions to the understanding of the creep processes.

**Graphical abstract:**

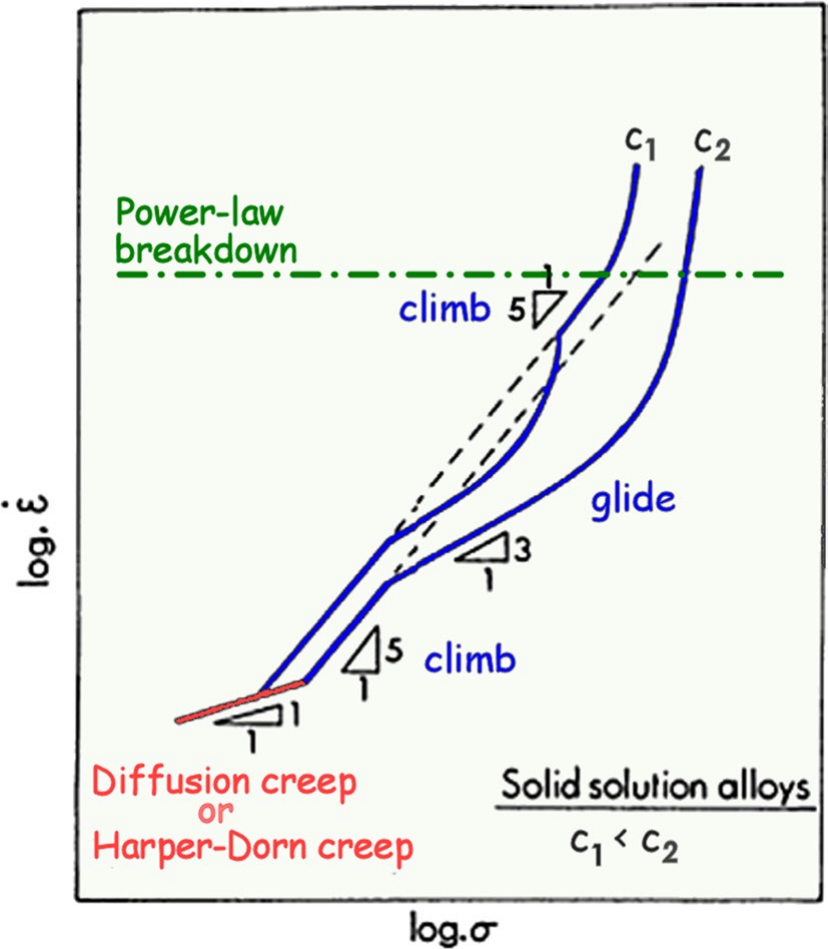

## Introduction

When a crystalline material is subjected to a very high stress it will fracture catastrophically but if the applied stress is lower than the fracture stress then the material will deform gradually in the process known as creep. The plastic strain introduced by creep is nonrecoverable and it builds up over an extended period of time. Creep processes are diffusion-controlled so that, although they occur at all temperatures above absolute zero, they become especially important at high temperatures, typically above ~ 0.4 *T*_m_, where *T*_m_ is the absolute melting temperature of the material.

**Figure 1:**
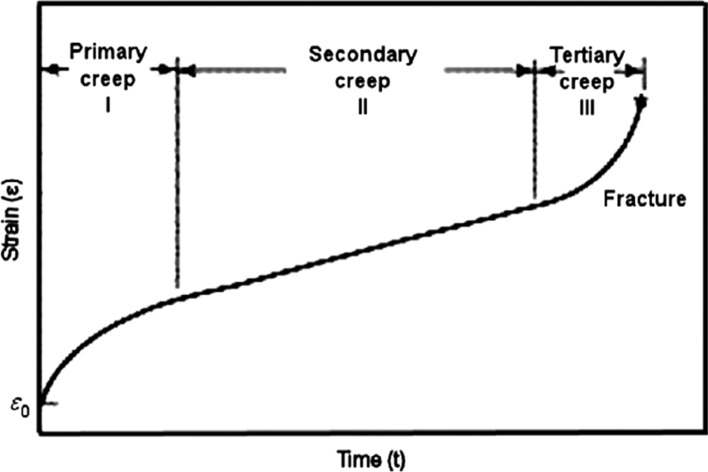
A representative creep curve showing the three regions of flow [1].

It is now well-established that creep occurs through the movement of defects within the crystalline lattice. In practice, these defects may be dislocations as in conventional dislocation creep where the flow process depends upon the nature of the dislocation movement or vacancies as in diffusional creep where vacancies move within the material either through the crystalline lattice or along the grain boundaries. In dislocation creep, which tends to be the most common process in a wide range of materials, the accumulation of strain with time may be represented as shown in Fig. 1 where the strain, *ε*, is plotted against the time, *t* [1]. Thus, there is an initial instantaneous strain, *ε*_0_, on application of the load, a region of primary creep (I) where the creep rate gradually decreases with time, a region of secondary or steady-state creep (II) where the creep rate remains essentially constant and a large strain is acquired and then a region of tertiary creep (III) where the creep rate rapidly accelerates to fracture. The movement of vacancies in diffusional creep also produces an increase in strain with time but without the occurrence of a region of primary creep.

Because of the general dominance of dislocation creep in real materials, much of the emphasis in laboratory creep experiments is devoted to measuring and interpreting the flow behaviour under conditions of steady-state creep in region II where this behaviour is especially important because it accounts for most of the strain acquired in modern operating systems.

## Historical background to experiments on creep

In the eighteenth and nineteenth centuries the small-scale industries that were operating at that time were constructed for performance at relatively low temperatures so that the occurrence of any flow by creep was neither considered in constructing the operating machinery nor was the advent of creep of any great practical significance. However, this changed significantly in the early part of the twentieth century when attempts were made to increase the operating temperatures, and therefore the overall efficiency, of industrial plants such as steam boilers. It is appropriate, therefore, that the first scientific report on the occurrence of creep was by Trouton and Rankine [2] in 1904 at the beginning of the twentieth century where this paper described the stretching (or straining under tensile conditions) and torsional loading of lead wires when subjected to loads beyond the elastic limit. Remarkably, in this first report on creep the authors obtained results that correspond very closely to the modern interpretation of creep strain as shown in Fig. 1. It is worthwhile, therefore, quoting directly from their paper and noting the very close correlation with modern experiments on high-temperature creep. Specifically, the authors stated as follows:

“The behaviour of wires or rods when stressed longitudinally or torsionally beyond the elastic limit is of a complicated nature. The general character of the effect observed on the application of such a stress is as follows. First there is an immediate effect followed by an increase with time. The latter initially may be considerable, but gradually diminishes with time to what appears to be a small constant amount. It is convenient to refer to the first as the primary strain, to the final constant rate as the viscous flow, and the remaining intermediary effect as the secondary strain. What follows refers mostly to the secondary strain.”

It is interesting to note that, even in these first experiments conducted in 1904, the authors recognized and placed a major emphasis on the strain incurred in the steady-state region II. Shortly thereafter in 1905, Phillips [3] published a report describing the creep of India rubber, glass and metal wires of Cu, Pt, Ag, Au, Fe and steel, and he reported that the behaviour of the metal wires was different to glass because “the creep caused a permanent extension and when the load was removed there was little, if any, slow creep back.”

The most comprehensive early experiments on the creep of metals were undertaken by Andrade and published in detailed reports in 1910 [4] and 1914 [5]. These experiments were performed initially on Pb and later on Sn, Fe, Cu, a Pb–Sn alloy and brass with the overall objective of determining a fundamental flow law for metals and developing a physical understanding of the flow process. Since these experiments were conducted before the concept of dislocations, the interpretation was necessarily different from a modern understanding of creep. Nevertheless, it is interesting to note that Andrade deduced that the variation of flow with time followed a *t*^1/3^ law and this was interpreted in terms of the existence of two different phases in the metals consisting of separate crystalline and amorphous phases. Although the concept of two distinct phases is no longer acceptable, the possibility that the creep strain is proportional to time raised to the power of 1/3 continues to attract attention and is examined even in recent publications [6–8].

## The flow mechanisms in creep deformation

Many of the creep experiments conducted over the last few decades have focussed on measuring the strain rates occurring in the steady-state or secondary region II as a function of various variables, such as stress, temperature and grain size, and then using these results to reach conclusions concerning the physical nature of the flow processes occurring within the crystalline matrix. It is now generally recognized that the steady-state creep rate, $\dot{\varepsilon },$ may be expressed through a simple relationship of the form [9, 10]

1 $$\dot{\varepsilon } = \frac{{{\text{ADGb}}}}{{{\text{kT}}}}\left( {\frac{{\text{b}}}{d}} \right)^{p} \left( {\frac{\sigma }{G}} \right)^{n},$$

where *D* is the diffusion coefficient that defines the creep flow mechanism and is equal to *D*_o_ exp (− *Q*/RT), where *D*_o_ is a frequency factor, *Q* is the activation energy for the diffusive process, *R* is the gas constant and *T* is the absolute temperature, *G* is the shear modulus of the material, **b** is the Burgers vector, *k* is Boltzmann’s constant, *d* is the grain size, *σ* is the applied stress, *p* and *n* are the exponents of the inverse grain size and the stress, respectively, and *A* is a dimensionless constant. A detailed inspection of Eq. (1) shows that, under any selected testing conditions, the creep rate will be defined exclusively by the values of the four parameters *Q*, *n*, *p* and *A*.

The basic principle of creep controlled by dislocation processes is based on the emission of dislocations from Frank-Read sources on different slip planes and the subsequent interactions between dislocations on different planes, the pile ups of edge dislocations on these planes and then, under the action of back stresses, the climbing together and annihilation of the leading dislocations. This process is generally controlled by the rate of dislocation climb, $\mathop {\dot{\varepsilon}_{c} },$ where a theoretical model shows that *Q* is equal to the value for self-diffusion, *n* has a value of the order of ~ 4.0 to 5.0 with the exact value dependent upon the value of the stacking fault energy and *p* is equal to zero because the process occurs intragranularly so that there is no dependence on grain size [11]. Conversely, in some solid solution alloys there may be a segregation of solute atoms around the moving dislocations to form Cottrell atmospheres so that glide becomes slower than climb and under these conditions the rate of creep by dislocation glide, $\mathop {\dot{\varepsilon}_{{\text{g}}} }$, is again given by Eq. (1) but with *Q* equal to the value for interdiffusion of the solute, *n* = 3 and again *p* = 0 [12].

For climb-controlled creep the creep rate is given by an expression of the form

2 $$\dot{\varepsilon }_{{\text{c}}} = A^{\prime } \phi \left\{ {\frac{\gamma }{{{\text{Gb}}}}} \right\}\frac{{{\text{DGb}}}}{{{\text{kT}}}}\left( {\frac{\sigma }{G}} \right)^{n},$$

where *γ* is the stacking fault energy and *n* is close to 5, and for glide-controlled creep the creep rate is given by an expression of the form

3 $$\dot{\varepsilon }_{{\text{g}}} = \user2{ }\frac{{\pi \left( {1 - {\upnu }} \right)kT\tilde{D}}}{{6e^{2} c{\mathbf{b}}^{5} G}}\left( {\frac{{{\sigma}}}{G}} \right)^{3},$$

where *ν* is Poisson’s ratio, $\widetilde{D}$ is the diffusion coefficient for the solute atom, *e* is the solute–solvent size difference and *c* is the concentration of the solute atoms.

**Figure 2:**
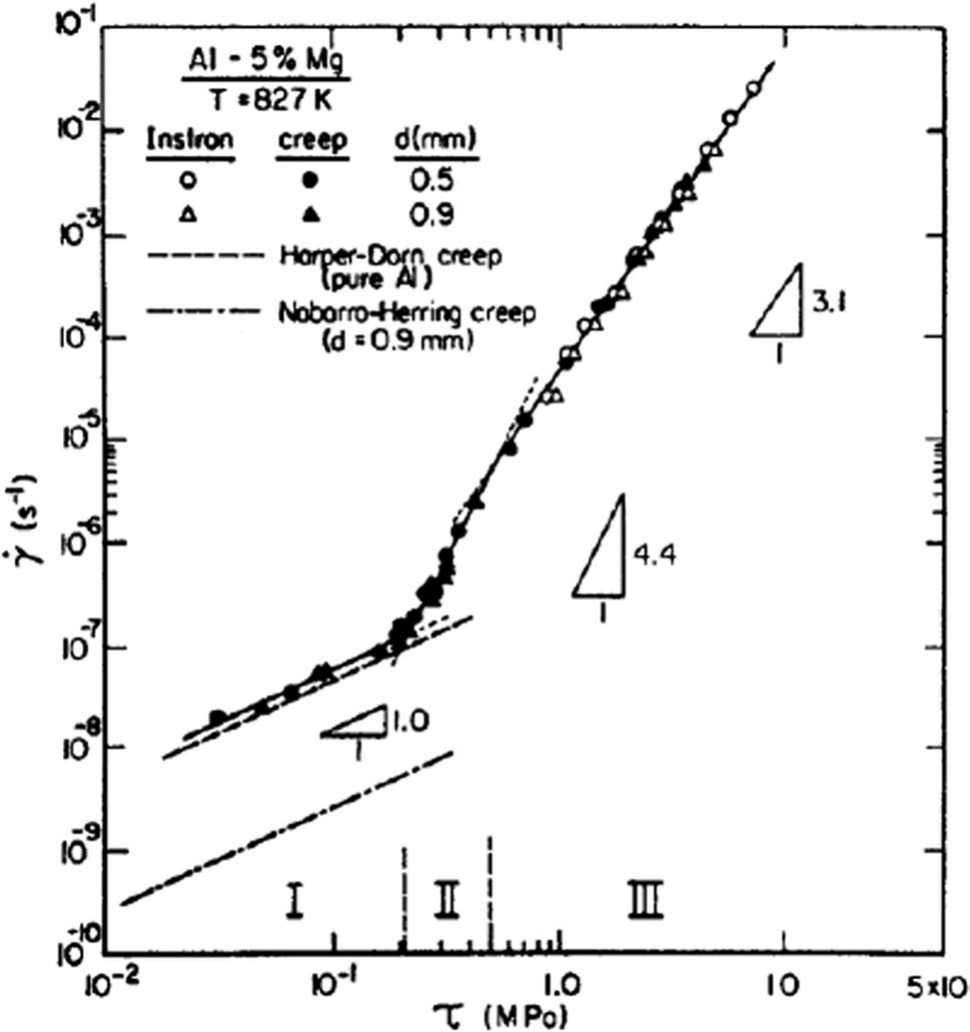
Shear strain rate versus shear stress for an Al-5% Mg alloy tested at 827 K showing the transitions between Harper-Dorn creep (I), dislocation climb (II) and dislocation glide (III) [13].

Figure 2 shows an example of high-temperature creep data for an Al-5% Mg alloy tested at 827 K under both creep conditions of constant stress and Instron conditions of constant strain rate using two different grains sizes of 0.5 and 0.9 mm [13]. The experimental points are shown as the shear strain rate, $\dot{\gamma }$, plotted against the shear stress, *τ*, where the points from both of these procedures superimpose but the data can be extended to lower stresses using creep and to higher stresses using Instron testing.

It is readily apparent that the datum points in Fig. 2 fall along a single line for both grain sizes so that the results are independent of grain size over the total range of experimental stress and this line divides into three separate regions which are designated from I to III. In region I, the stress exponent is *n* = 1.0 with the results almost superimposing on earlier results reported for Harper-Dorn creep in pure Al where this creep was predicted from very early creep experiments [14] and the dashed line in Fig. 2 is based on a detailed analysis of the creep of Al at very low stress levels [15]. Harper-Dorn creep has been documented and evaluated in numerous publications on high-temperature creep and is generally recognized as a dislocation-controlled mechanism having a linear dependence on the applied stress [16–23]. The lower dashed line in region I is the prediction for Nabarro-Herring diffusional creep for a grain size of 0.9 mm in pure Al [24, 25] and this line clearly deviates significantly from the experimental results for the Al-5% Mg alloy so that region I is unambiguously attributed to the occurrence of Harper-Dorn creep.

At higher stresses, the short region II has a stress exponent of *n* ≈ 4.4 suggesting creep controlled by conventional dislocation climb and in region III there is a stress exponent of *n* ≈ 3.1 suggesting control by dislocation glide. The transition from climb to glide is also consistent with substructural observations showing a change from subgrain formation to a random array of dislocations as the stress increases from region II to region III.

**Figure 3:**
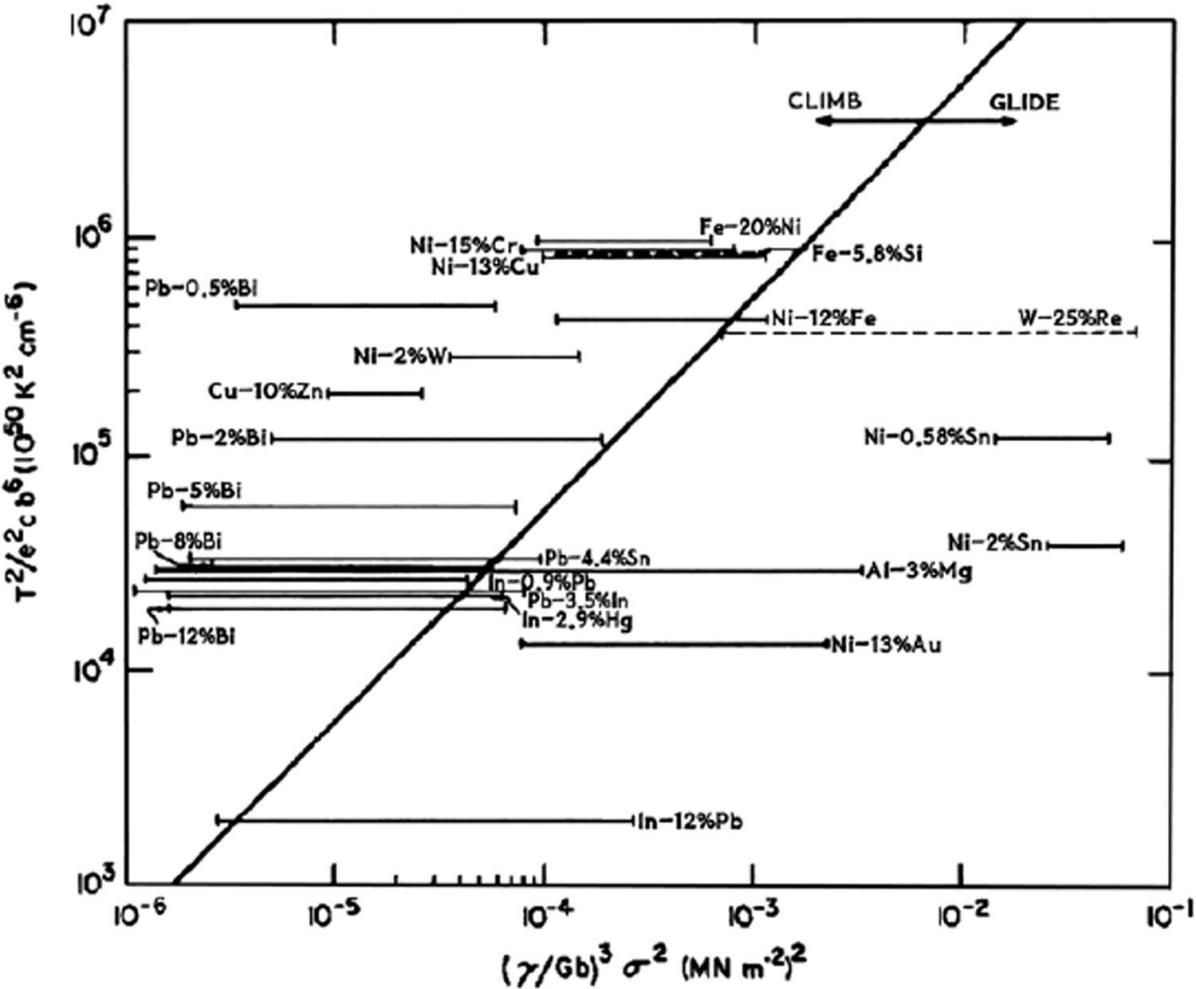
Predicted transition between dislocation climb (on left) and viscous glide (on right) in metallic solid solution alloys [26].

Using creep data of this type, and the fundamental rate equations for control by dislocation climb and dislocation glide, it is possible to derive a relationship for the magnitude of the applied stress that marks the transition from climb to glide in creep experiments. This approach leads to the following condition for the occurrence of viscous glide in high-temperature creep [26]:

4 $$\frac{B{\sigma }^{2}}{{k}^{2}(1-\upsilon )}{\left(\frac{\gamma }{G\mathbf{b}}\right)}^{3}>\frac{{T}^{2}}{{e}^{2}c{\mathbf{b}}^{6}},$$

where *B* is a constant having a predicted value of ~ 8 × 10^12^. Equation (4) is shown in Fig. 3, where the solid line at 45° separates alloys deforming by dislocation climb on the left from alloys deforming by dislocation glide on the right with results shown for a large number of materials based on published data as summarized earlier [26]. In this plot, all alloys have a face-centred cubic structure except Fe-5.8% Si and W-25% Re which are body-centred cubic and are shown in Fig. 3 using dashed lines. A careful examination of many alloys using this climb-glide criterion showed excellent agreement with all experimental alloys except only for Au-10% Ni which is not shown in Fig. 3 but this alloy lay on the wrong side of the boundary because of uncertainty in the precise value of the stacking fault energy.

**Figure 4:**
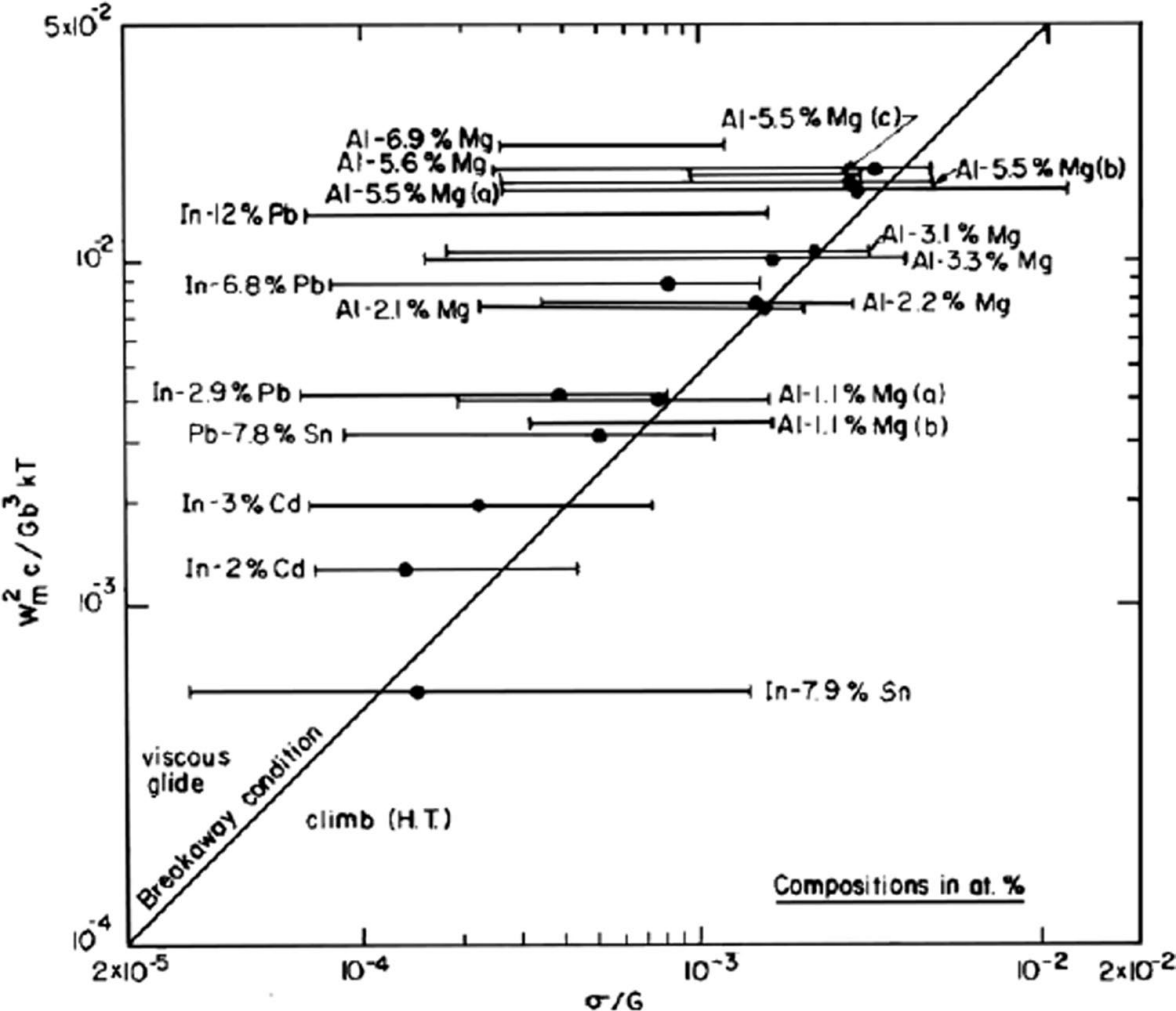
Predicted breakaway from viscous glide at high stresses in solid solution alloys [27].

Extensive experiments show that the creep behaviour of solid solution alloys is even more complicated because the stress exponent increases above a value of *n* ≈ 3 at very high stress levels, where this is attributed to the ability of dislocations to break away from their solute atom atmospheres at these stress levels. It was shown theoretically that there is a critical breakaway stress, *σ*_b_, which is given by a relationship of the form [27].

5 $${\sigma }_{\text{b}}=\frac{{W}_{m}^{2}c}{5{\mathbf{b}}^{3}kT},$$

where *W*_m_ is the binding energy between the solute atmosphere and the dislocation. Figure 4 shows a plot of this relationship where both terms were divided by *G* in order to provide values for the normalized stress on the abscissa. Thus, the line at 45° corresponds to the breakaway condition denoting a transition from viscous glide with *n* ≈ 3 at the lower stresses to a transition region with *n* > 3 and ultimately to conventional high-temperature (HT) dislocation climb with *n* ≈ 5 at the highest stresses. Superimposed on Fig. 4 are the results from published data for various solid solution alloys, where the solid circle on each line denotes the value of the normalized stress reported experimentally for breakaway so that at higher stresses the stress exponent becomes larger than ~ 3. It is readily apparent that the theoretical line is in excellent agreement with the experimental results for all alloys, including for an Al-6.9% Mg alloy [28] and an In-12% Pb alloy [29] where there was no evidence for any breakaway from the region controlled by dislocation glide.

**Figure 5:**
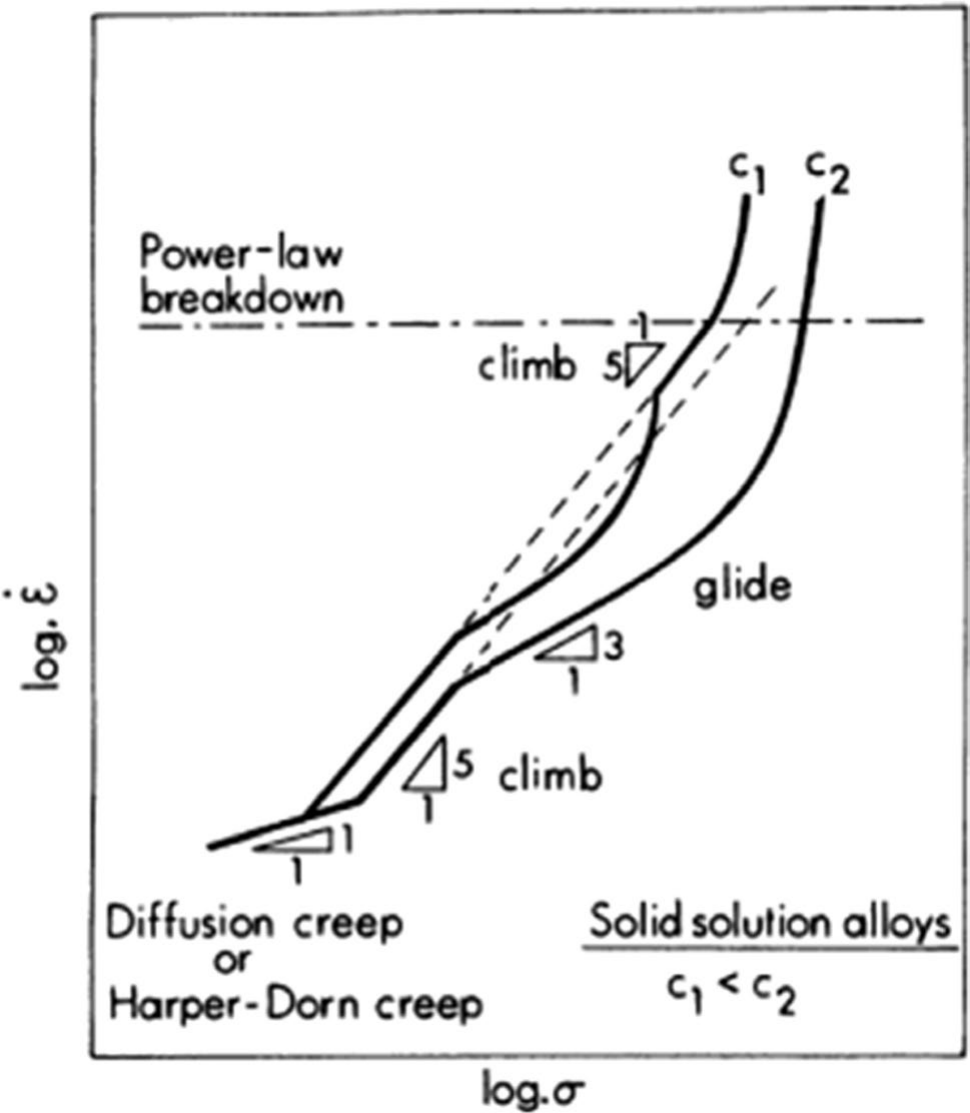
Schematic illustration of strain rate versus stress for solid solution alloys showing the effect of solute concentration [30].

Based on these analyses, it is possible to schematically illustrate the anticipated trends associated with the creep of solid solution alloys as shown in Fig. 5, where the strain rate, $\dot{\varepsilon }$, is logarithmically plotted against the stress, *σ* [30]. Predictions are illustrated for two different solute concentrations, *c*_1_ and *c*_2_, where the concentration *c*_1_ is lower than the concentration *c*_2_. Thus, in *c*_2_ at the highest concentration there are transitions from a region at the lowest stresses with *n* = 1 due to diffusional creep or Harper-Dorn creep, a region with *n* ≈ 5 due to dislocation climb, a transition into an extended region of *n* ≈ 3 due to control by dislocation glide and then a break leading to conventional power-law breakdown at the highest stresses and fastest strain rates. Conversely, for the lower concentration *c*_1_ there is a similar variation with stress except that the glide region is shorter and there is an additional region of climb with *n* ≈ 5 occurring immediately prior to power-law breakdown.

**Figure 6:**
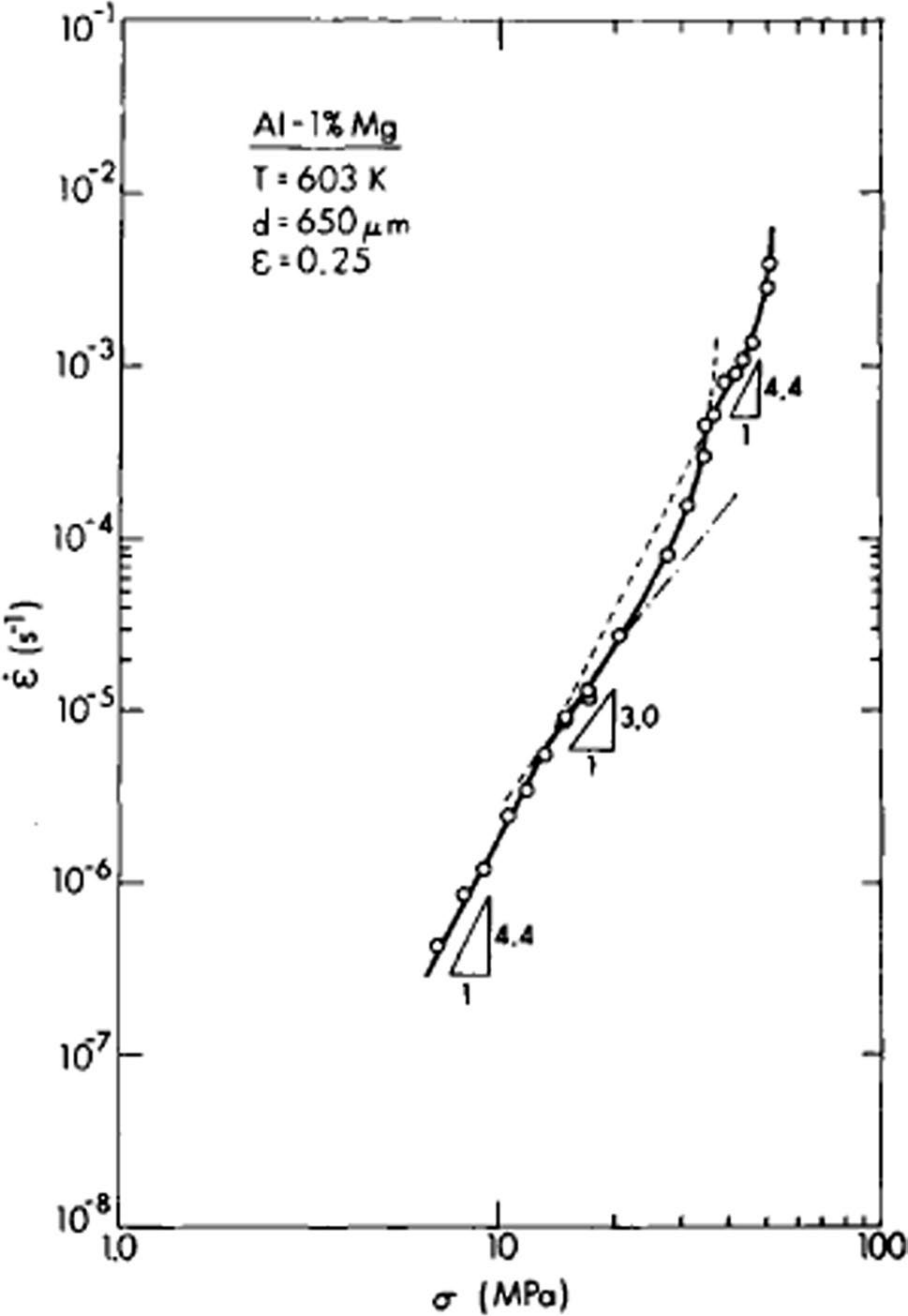
Steady-state creep rate versus stress for Al-1% Mg at a temperature of 603 K [31].

It is apparent from Fig. 5 that at very low solute concentrations there may be a second climb region occurring at the highest stress levels immediately before the power-law breakdown. An example of this second climb region is shown in Fig. 6 where an Al-1% Mg alloy was creep tested at 603 K with a grain size of 650 µm [31]. The results show that at a stress of ~ 35 MPa there is a transition to a slope of *n* = 4.4 which corresponds to an extrapolation from the regions of dislocation glide and breakaway at lower stresses and then at a stress of ~ 47 MPa there is a clear upwards swing representing the transition into the region of power-law breakdown.

## The significance of grain boundaries in creep deformation

Intragranular creep processes become of special importance when the grain size is small. For example, the principle of diffusional creep is based on the understanding that, under the action of an external stress, vacancy concentrations are enhanced on those grain boundaries lying more nearly perpendicular to the tensile axis whereas the concentrations are depleted on those grain boundaries lying more nearly parallel to the tensile axis [32]. This produces a stress-directed vacancy flow which may occur either through the crystalline lattice in Nabarro-Herring diffusion creep [24, 25] or along the grain boundaries in Coble diffusion creep [33].

When testing materials to determine the flow properties, it is possible to use conventional creep testing where the material is subjected to a constant stress and the measured creep rate is then plotted against the stress using logarithmic coordinates in order to determine the stress exponent, *n*, as defined by the relationship given in Eq. (1). Alternatively, tests may be conducted using an Instron machine to apply a constant strain rate so that the flow stress is measured and the stress and strain rate are then related through an expression of the form

6 $$\sigma = B_{1} \dot{\varepsilon }^{m},$$

where *m* is the strain rate sensitivity which is equal to 1/*n* and *B*_1_ is a constant incorporating the dependence on temperature and grain size. An example was shown earlier in Fig. 2 to demonstrate the equivalence between these two types of testing.

An important creep mechanism associated with the presence of grain boundaries is grain boundary sliding (GBS) whereby individual grains are displaced relative to each other with the displacements occurring at the grain boundaries. Superplasticity refers to the potential for achieving exceptionally high strains when a material is pulled in tension and, specifically, superplasticity is defined as an elongation of at least 400% and a measured strain rate sensitivity close to ~ 0.5 which is equivalent to a stress exponent of ~ 2 [34]. It is now well-established that superplastic flow requires a testing temperature that is generally above ~ 0.5 *T*_m_ and also it requires a very small grain size that is typically below ~ 10 µm [9].

An important initial requirement in studying superplastic flow was to obtain a careful measure of the value of *n* associated with the occurrence of superplasticity. This objective was made difficult because the earliest experiments suggested that superplastic flow occurred over a limited range of intermediate strain rates with a strain rate sensitivity close to ~ 0.5 but with a lower strain rate sensitivity when testing at faster strain rates. The situation at strain rates below the superplastic range was not well-defined in these early experiments because there were reports of both an increase and a decrease in the value of *m* at these lowest strain rates. The main problem associated with these earlier experiments was that they were conducted by using a single specimen and then pulling at different strain rates to measure the respective flow stresses without changing the sample. This approach has the advantage that it is easy and relatively quick but it has a major disadvantage because it fails to incorporate the grain growth which will typically occur when testing at elevated temperatures and therefore the results do not represent the data that would be obtained at a single well-defined grain size.

**Figure 7:**
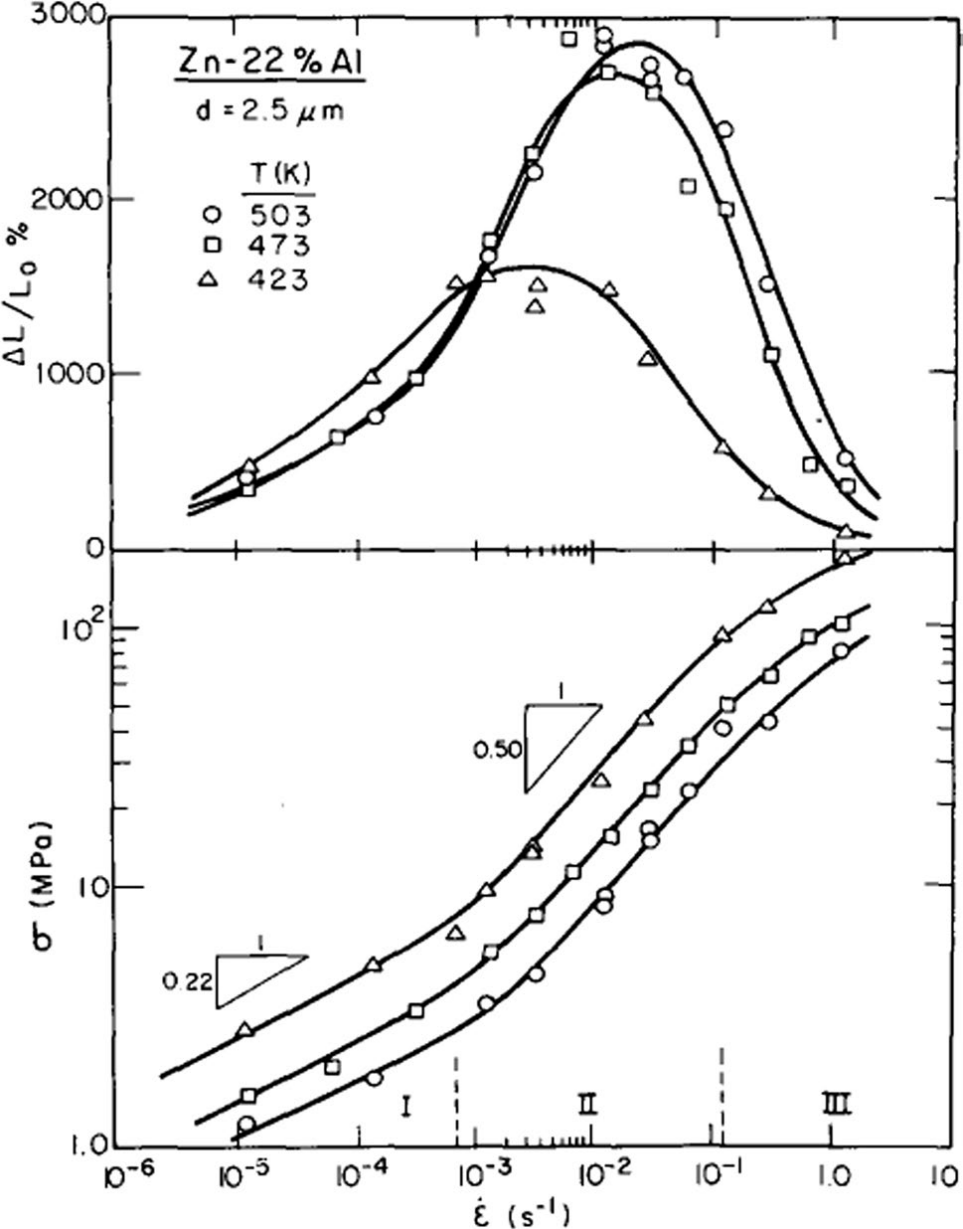
Variation of elongation to failure (upper) and flow stress (lower) with strain rate for a Zn-22% Al alloy with a grain size of 2.5 µm tested at three different temperatures [36].

This problem may be overcome by using a series of specimens having identical grain sizes and then testing each specimen to failure at a different strain rate and carefully measuring the associated flow stress and elongation. The first experiment using this procedure showed unambiguously that the results followed a sigmoidal curve for a Zn-22% Al eutectoid alloy with a grain size of 2.5 µm when plotting stress against strain rate at a temperature of 473 K [35] and later the results were expanded to cover the same material when testing at 423 and 503 K [36]. These latter results are shown in Fig. 7 with the flow stress plot against the strain rate (lower) depicting a sigmoidal type of behaviour of three regions, labeled I, II and III, with *m* ≈ 0.22 at the lowest strain rates, ~ 0.50 at intermediate strain rates and then again a reduction in *m* at the highest strain rates. Plotting the elongations to failure, Δ*L*/*L*_o_%, against the strain rate (upper), where Δ*L* is the increase in length and *L*_o_ is the initial gauge length, leads to a well-defined series of bell-shaped curves with exceptionally high elongations, up to > 2000% at the two highest temperatures, and much reduced elongations at the slower and faster strain rates. These high elongations represent true superplastic flow and they are associated with a measured strain rate sensitivity of *m* ≈ 0.5. The experimental results, therefore, firmly establish the decrease in the strain rate sensitivity at low strain rates and the requirement of a stress exponent of *n* ≈ 2 for superplasticity.

An important requirement in these types of experiments is to determine the rate-controlling flow process and specifically the range of experimental conditions associated with each flow mechanism. This may be determined by plotting deformation mechanism maps as first developed in diagrams showing, for a selected grain size, the normalized stress plotted on a logarithmic axis against the homologous temperature, *T*/*T*_m_ [37]. However, a limitation of these diagrams was that they were difficult to construct since the boundaries between the separate flow mechanisms were given by curved lines and this involved extensive computation. Furthermore, each diagram applied only to the selected grain size and significant calculations were needed to construct a similar map for the same material having a different grain size. Accordingly, the approach was changed to plot the normalized grain size, *d*/**b**, against the normalized stress, *σ*/*G*, on logarithmic axes for any selected temperature and in this form the boundaries separating the flow mechanisms are straight lines and it is an easy process to then construct a new map for a different testing temperature [38].

**Figure 8:**
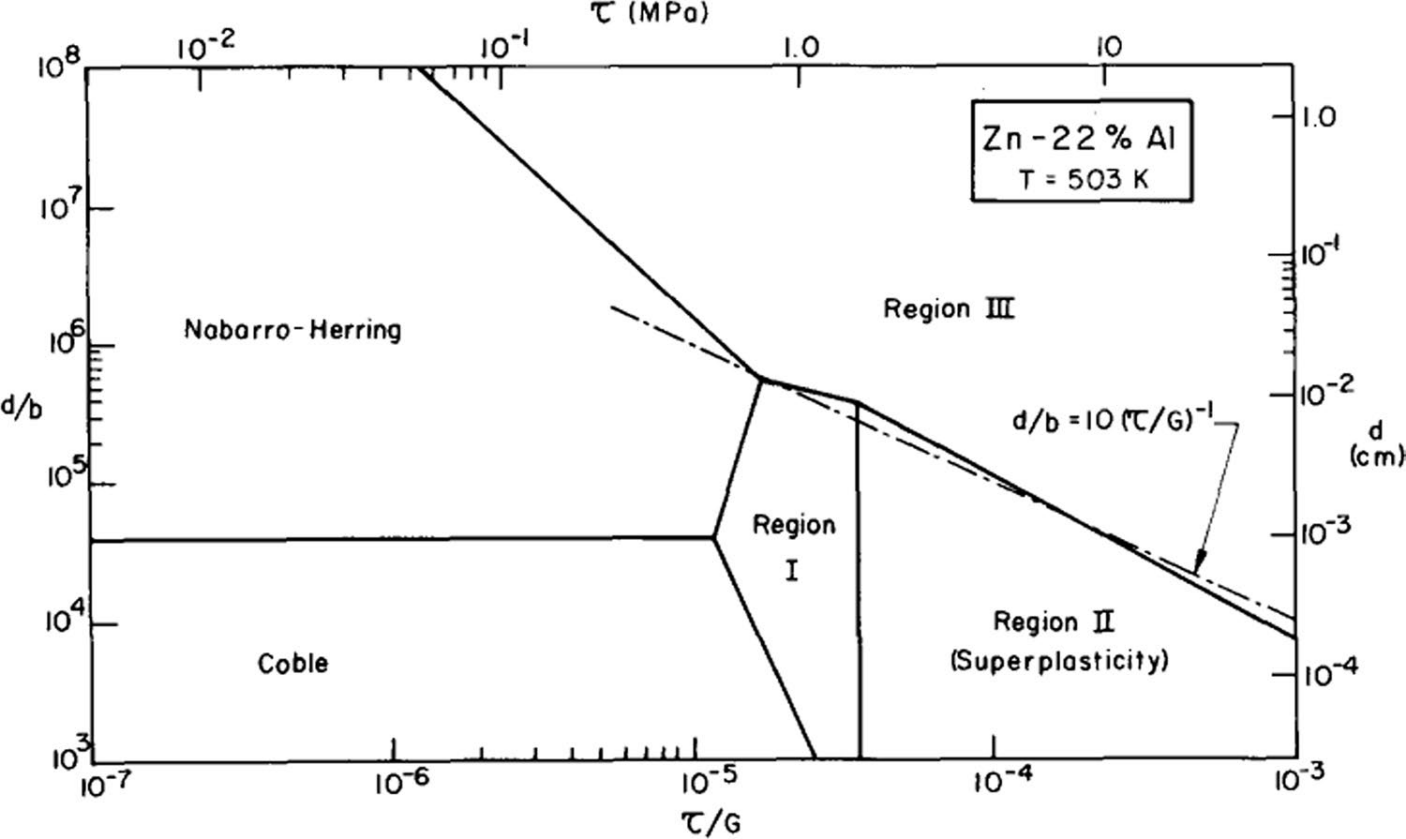
Deformation mechanism map for Zn-22% Al at 503 K, where the dashed line denotes the lower limiting grain size for the formation of subgrains [39].

An example of this type of map is shown in Fig. 8 using experimental data taken from Fig. 7 for the Zn-22% Al eutectoid alloy when testing at a temperature of 503 K and plotting the results as regions I, II (superplasticity) and III together with the theoretical predictions for the flow mechanisms of Nabarro-Herring [24, 25] and Coble [33] diffusion creep and plotting the stress in terms of the shear stress, *τ* [39]. In Fig. 8 each flow mechanism is represented by a field in grain size-stress space and the field boundaries delineate the transitions to a different rate-controlling flow mechanism.

Under conventional creep conditions for non-superplastic materials having large grain sizes it is well-established that subgrains are formed within the grains during the creep process. From an analysis of data from several creep experiments, it was established that the average size of the subgrains, *λ*, may be expressed through a relationship of the form [40]

7 $$\frac{\lambda }{{\varvec{b}}}= {B}_{2}{\left(\frac{\uptau }{G}\right)}^{-1},$$

where *B*_2_ is a constant having a value of ~ 10. This type of relationship is now recognized as generally applicable for the creep of metals [41] and the same relationship was reported for ceramic materials [42] and there are also reports of a relationship of this form in the deformation of rocks and minerals [43–46]. Assuming that the grain size of the material is equal to the equilibrium subgrain size, it is feasible to superimpose the prediction of Eq. (7) onto the deformation mechanism map shown in Fig. 8. The result is given by the dashed line which corresponds remarkably closely with the field boundary separating the superplastic region II and the high strain rate region III. This plot demonstrates, therefore, that superplastic flow requires not only a small grain size but specifically a grain size that is smaller than the equilibrium subgrain size as given by Eq. (7).

**Figure 9:**
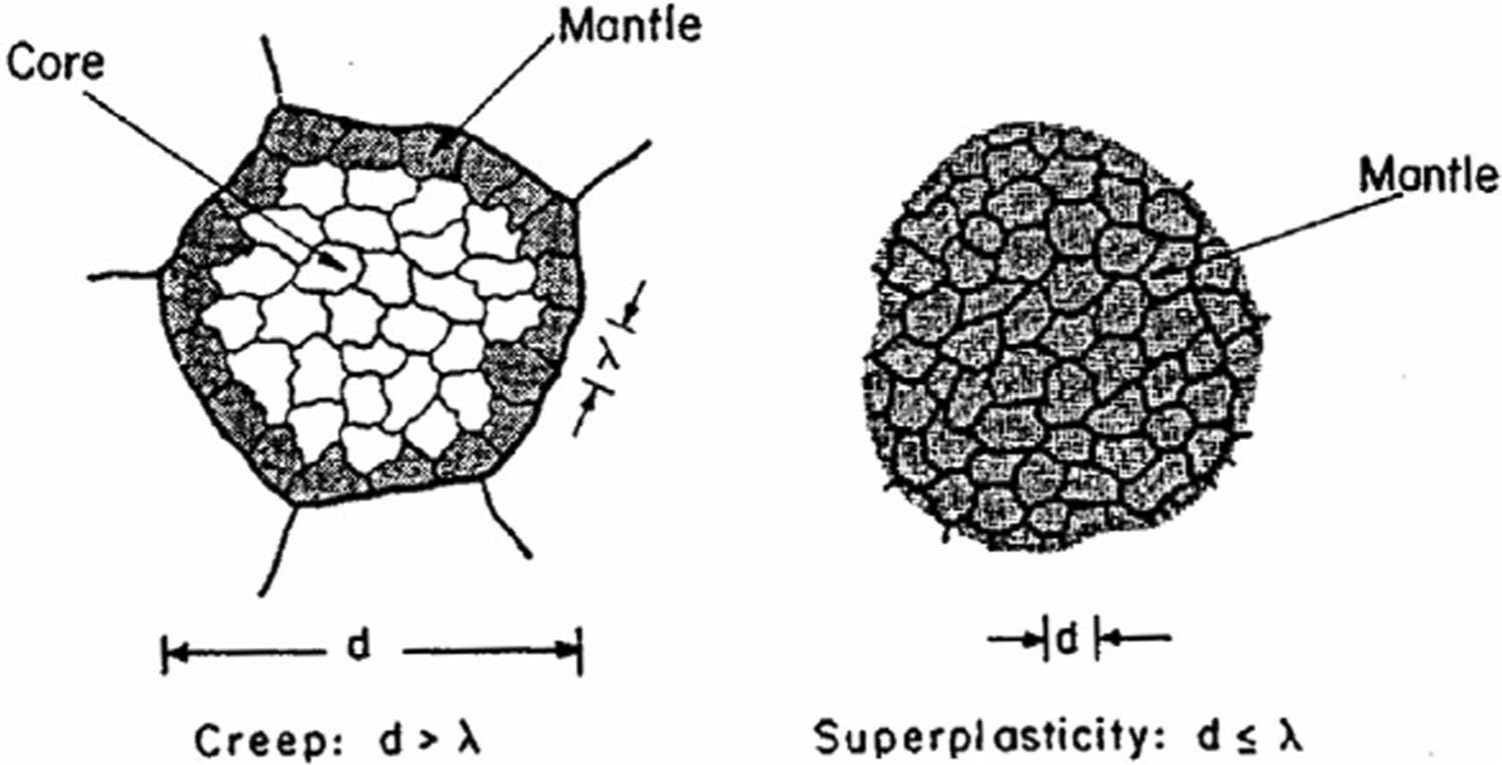
Schematic illustration of **a** creep with a large grain size (*d* > *λ*), where the grain is divided into a central core and a peripheral mantle of subgrains and **b** superplasticity with a small grain size (*d* ≤ *λ*), where subgrains are not formed and the grains represent mantle behaviour [47].

This result may be represented pictorially as shown in Fig. 9, where creep at large grain sizes is shown on the left so that *d* > *λ* and each grain becomes divided into two distinct parts so that there is an inner core comprised of subgrains and an outer mantle of subgrains around the edge of the grain [47]. By contrast, it is now apparent that superplasticity occurs when *d* < *λ* so that the inner core is absent and all of the specimen consists of a mantle of subgrains so that the deformation now corresponds to mantle-like flow.

A careful analysis of data shows that the flow process of GBS accounts for essentially all of the deformation occurring in superplasticity [48]. Several methods are available for measuring the contribution of GBS to the overall strain but it is important to note that measurements of the changes in grain shape [49, 50] are not a viable option because in high-temperature flow the occurrence of grain boundary migration tends to spheroidize the grains during creep and thereby produce an unrealistically high apparent value for the sliding contribution. This problem was demonstrated by comparing measurements of grain shape with the strains calculated by measuring the distortions of grids photographically printed within the grains on the surfaces of creep specimens [51].

**Figure 10:**
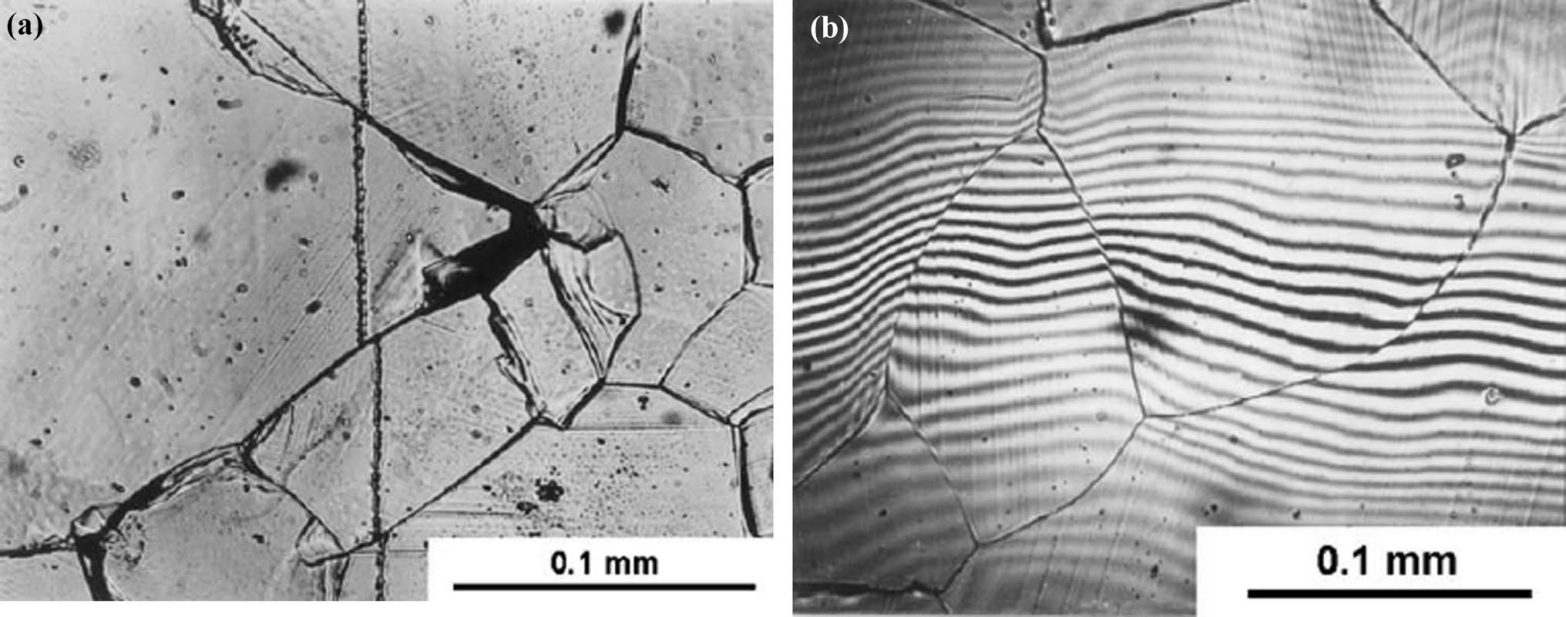
Evidence for grain boundary sliding in a Mg-0.78% Al alloy (**a**) with a transverse marker line revealing offsets in the plane of the specimen surface [52] and **b** using interferometry to reveal vertical offsets perpendicular to the specimen surface [53].

Several methods are now available for measuring the contribution from GBS during polycrystalline flow and two simple methods are shown in Fig. 10. First, transverse markers were scribed prior to creep on the surface of a sample of an Mg-0.78% Al alloy with a grain size of 95 µm and then the specimen was pulled in tension to a strain of ~ 2.5% at a temperature of 473 K [52]. The result is shown in Fig. 10a, where the stress axis is horizontal and there are sharp and measurable offsets in the marker line at the points where it crosses the grain boundaries and with the offsets lying in the plane of the surface. Second, it is possible to use interferometry to measure the vertical offsets at the grain boundaries as shown in Fig. 10b, where the interferometric lines have a separation of 0.27 µm and there are clear displacements in the fringes at the points where they cross the grain boundaries [53]. In Fig. 10b the material is the same as in Fig. 10a and the sample was pulled in tension to a strain of ~ 1.5% at a temperature of 473 K.

## Examining the mechanism of GBS in creep and superplastic flow

**Figure 11:**
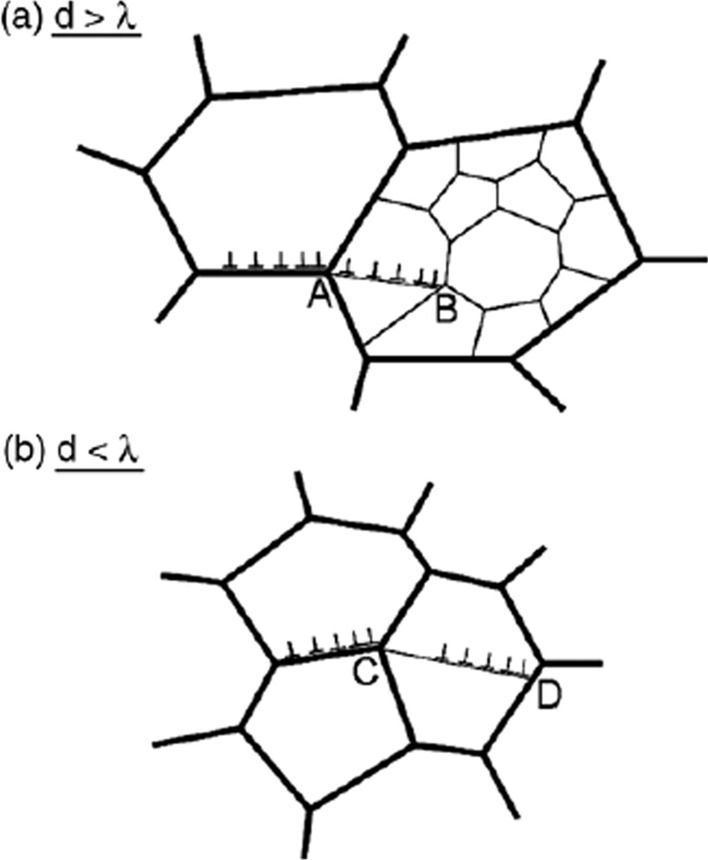
Schematic illustration of the principles of grain boundary sliding in **a** creep where subgrains are present so that there is a dislocation pile up at a subgrain boundary and **b** superplasticity where there are no subgrains and the dislocations pile up against the opposite grain boundary [55].

It is readily apparent from Fig. 9 that there is a significant difference between the flow behaviour under conventional creep conditions when the grains are reasonably large and under superplastic conditions when the grains are very small. An early model of GBS recognized the contribution from dislocations moving at the grain boundaries [54] but this model was developed before there was an understanding of the two separate flow processes of creep and superplasticity. Accordingly, the model was further developed with GBS occurring though dislocation movement along the boundaries and then a pile up creating a stress concentration at the next triple point as shown in Fig. 11 where (a) is for creep where *d* > λ and (b) is for superplasticity where *d* < λ [55]. The two relevant triple points are labeled A and C and in creep the stress concentration nucleates dislocation flow in the next grain so that the dislocations pile up at the first subgrain boundary labeled B whereas in superplasticity there are no subgrain boundaries and the dislocations cross the next grain and pile up at the opposing grain boundary at the point labeled D. An analysis shows that these two processes lead to different equations for GBS because in (a) flow is controlled by the climb of dislocations into the subgrain boundary whereas in (b) the dislocations climb into the grain boundary.

The rate of GBS under creep conditions, ${\dot{\varepsilon }}_{\text{gbs}},$ is given by the relationship [55]

8 $${\dot{\varepsilon }}_{\text{gbs}}=\boldsymbol{ }\frac{{A}_{\text{gbs}}{D}_{{\ell}}G\mathbf{b}}{kT}\left(\frac{\mathbf{b}}{d}\right){\left(\frac{\upsigma }{G}\right)}^{3},$$

where *D*_ℓ_ is the coefficient for lattice self-diffusion, *A*_gbs_ is a constant having a value of ~ 10.^3^ and the stress exponent is *n* ≈ 3. Alternatively, the rate of GBS in superplasticity, ${\dot{\varepsilon }}_{\text{sp}}$, is given by [55]

9 $${\dot{\varepsilon }}_{\text{sp}}=\boldsymbol{ }\frac{{A}_{\text{sp}}{D}_{\text{gb}}G\mathbf{b}}{{kT}}{\left(\frac{\mathbf{b}}{d}\right)}^{2}{\left(\frac{\sigma }{G}\right)}^{2},$$

where *D*_gb_ is the coefficient for grain boundary diffusion, *A*_sp_ is a constant having a value of ~ 10 and both of the exponents *n* and *p* are equal to ~ 2. Equations (8) and (9) demonstrate, therefore, the change in the stress exponent for GBS depending on the precise nature of the accommodation for sliding.

The theoretical model relating to Eq. (9) was derived specifically for deformation occurring at elevated temperatures because it incorporates an approximation that very rapid diffusion occurs at the operating temperatures so that there is little or no development of a supersaturation of vacancies. This approach may be modified to cover flow at lower temperatures by simply removing this approximation and instead permitting the build-up of a supersaturation. Under these conditions, the GBS occurring at low temperatures is then given by a modification of the basic equation of the form [56].

10 $${\dot{\varepsilon }}_{\text{sp}(\text{LT})}\approx \frac{10\delta {D}_{\text{gb}}}{{d}^{3}}\left[\text{exp}\left(\frac{2d{\sigma }^{2}{\mathbf{b}}^{2}}{3{GkT}}\right)-1\right],$$

where LT denotes low temperatures and *δ* is the grain boundary width which is generally approximated as equal to 2*b*. This means in practice that at low temperatures and high stresses the strain rates produced are faster through Eq. (10) than through Eq. (9). Finally, it is necessary to note that Eq. (10) no longer predicts a constant strain rate sensitivity but rather the individual experimental values of m may be lower than the conventional value of 0.5. A more detailed analysis of this approach was presented in earlier reports [57, 58].

## The potential for making a direct comparison between flow data and theoretical creep mechanisms

It follows from the preceding discussion that the flow mechanisms in high-temperature creep are now understood reasonably well. Specifically, there may be Nabarro-Herring diffusion creep [24, 25], Coble diffusion creep [33] or Harper-Dorn creep [14] at low stress levels, creep by dislocation climb [11] or dislocation glide [12] at higher stresses and grain boundary sliding at the larger grain sizes in creep conditions and grain boundary sliding at small grain sizes in superplasticity [55, 59]. The transitions associated with the dislocation processes are also now well documented [26, 27]. Therefore, it is relatively easy to make direct comparisons between experimental data and the predicted creep behaviour. There are numerous reports undertaking these types of comparisons and in this section three different examples are considered.

First it is necessary to note an important development over the last 30 years where it was demonstrated that it is feasible to produce polycrystalline metals with exceptionally small grain sizes, typically within the submicrometer range and often at the nanometer level, by subjecting the large-grained metals to various processes involving the application of severe plastic deformation (SPD). This effect was first reported in 1988 [60] and subsequently, as the processing was further developed, it was the subject of several reviews [61–64]. There are a number of different procedures for subjecting a material to SPD to achieve a refining of the grain size but the two main processes are equal-channel angular pressing (ECAP) where a rod or bar is pressed through a die constrained within a channel that is bent through an abrupt angle within the die [65] and high-pressure torsion (HPT) where the sample, generally in the form of a thin disc, is subjected to a high applied pressure and concomitant torsional straining [66]. Several very recent reviews are now available summarizing the more significant developments in the field of SPD processing [67–71].

**Figure 12:**
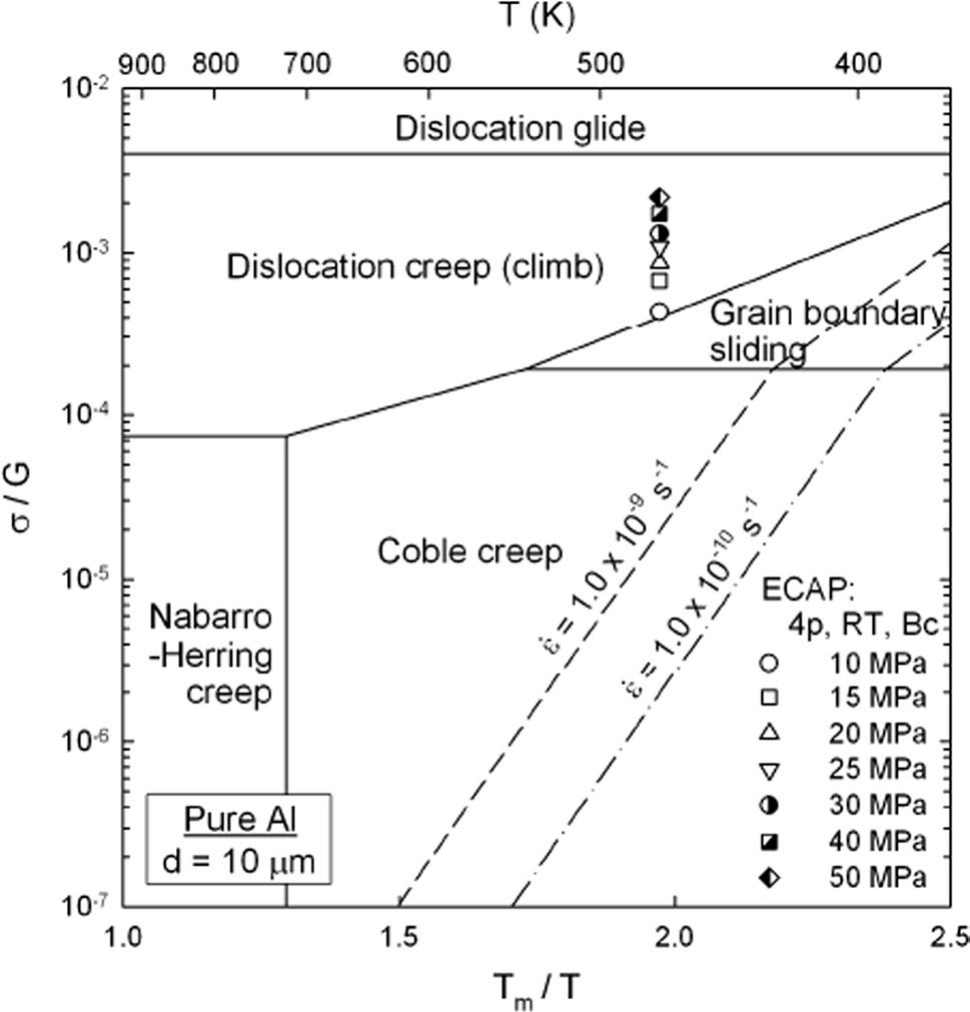
Deformation mechanism map for pure Al with a grain size of ~ 10 μm showing experimental datum points and two strain rate contours [73].

As a first example of a comparison between experimental data and theoretical predictions, rods of pure Al were processed by ECAP to reduce the grain size from an initial value of ~ 1 mm to a final value of ~ 1.3 µm after 4 passes of ECAP at room temperature (RT) using processing route *B*_C_ where the rod is rotated by 90° around the longitudinal axis between each pass. The pressed material was then subjected to creep testing at 473 K using constant stresses from 10 to 50 MPa and the creep rates were recorded within the steady-state regions. Based on earlier creep results for pure Al, it is known that the grains grow rapidly in the early stages of deformation so that the sizes in the steady-state region are of the order of ~ 10 to 12 µm [72]. Assuming the grains grow by about one order of magnitude to ~ 10 µm, the experimental creep results may be superimposed on a deformation mechanism map of the normalized stress against the inverse of the homologous temperature as shown in Fig. 12 [73] where this type of map was first proposed as a simple alternative to the conventional maps because the field boundaries now appear as straight lines [74]. Thus, the map depicts the locations of the Nabarro-Herring [24, 25] and Coble [33] creep processes, grain boundary sliding using Eq. (9) because of the small grain size and the regions of dislocation climb and glide. The map includes two contours of constant strain rate corresponding to $1.0 \times 10^{ - 9} \;{\text{and}}\;1.0 \times 10^{ - 10} \;{\text{s}}^{ - 1}$ since these represent essentially the lower limiting strain rates for recording meaningful creep measurements in laboratory tests. The experimental datum points recorded in these experiments are shown in Fig. 12 and they all lie within the field of dislocation climb, where this is consistent with the experimental data since the measured stress exponent in the steady-state region was $n \approx 5$. Thus, the theoretical predictions are in excellent agreement with the experiments.

**Figure 13:**
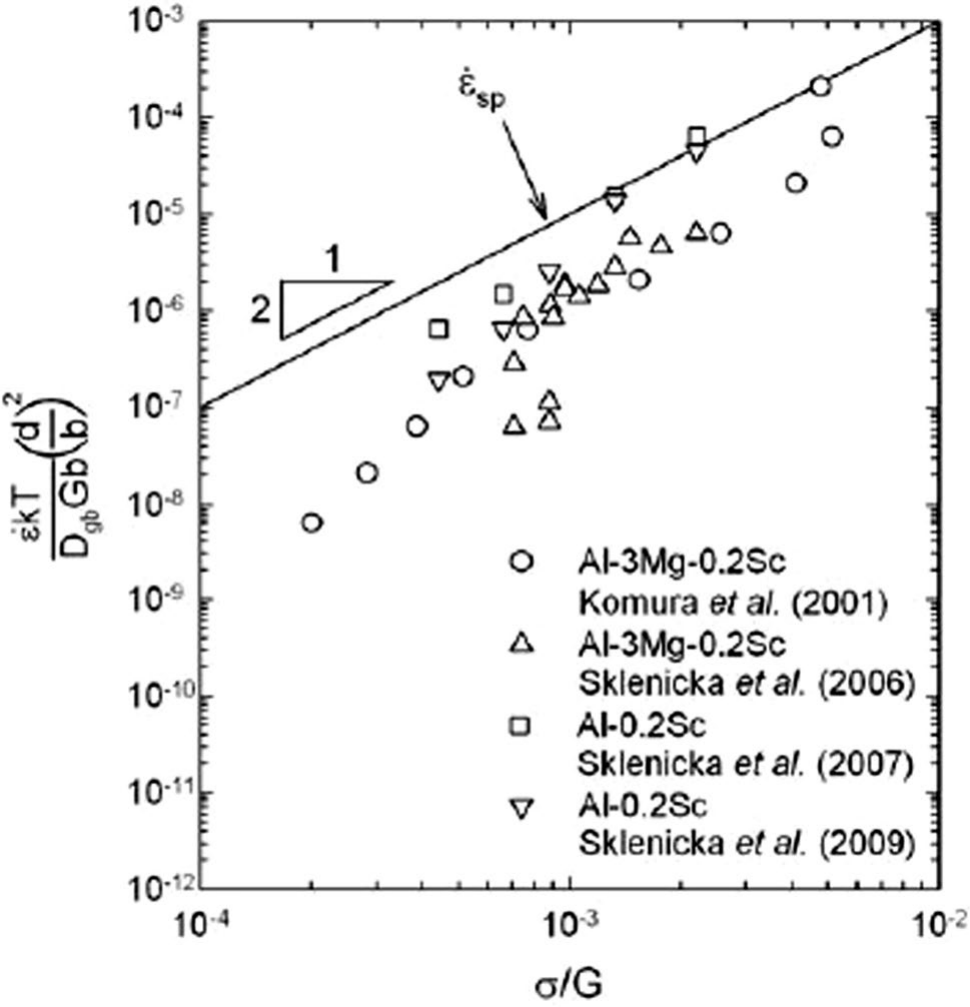
Temperature and grain size compensated creep rate plotted against the normalized stress showing all experimental datum points [72, 76–78] and the predicted behaviour for conventional superplasticity [75].

As a second example, it is generally usual in SPD materials to compare the measured normalized strain rates with the predictions from Eq. (9). An example is shown in Fig. 13, where the upper solid line denotes ${\dot{\varepsilon }}_{\text{sp}}$ in a plot of the temperature and grain size compensated creep rate plotted against the normalized stress [75]. Superimposed on this plot are datum points estimated from various creep experiments on Al–Mg–Sc and Al–Sc alloys [72, 76–78]. This plot shows there is excellent agreement with the experimental data for the Al-0.2% Sc alloy and for the Al-3% Mg-0.2% Sc alloy most of the experimental points lie within an order of magnitude of the theoretical prediction thereby confirming the overall applicability of this procedure.

**Figure 14:**
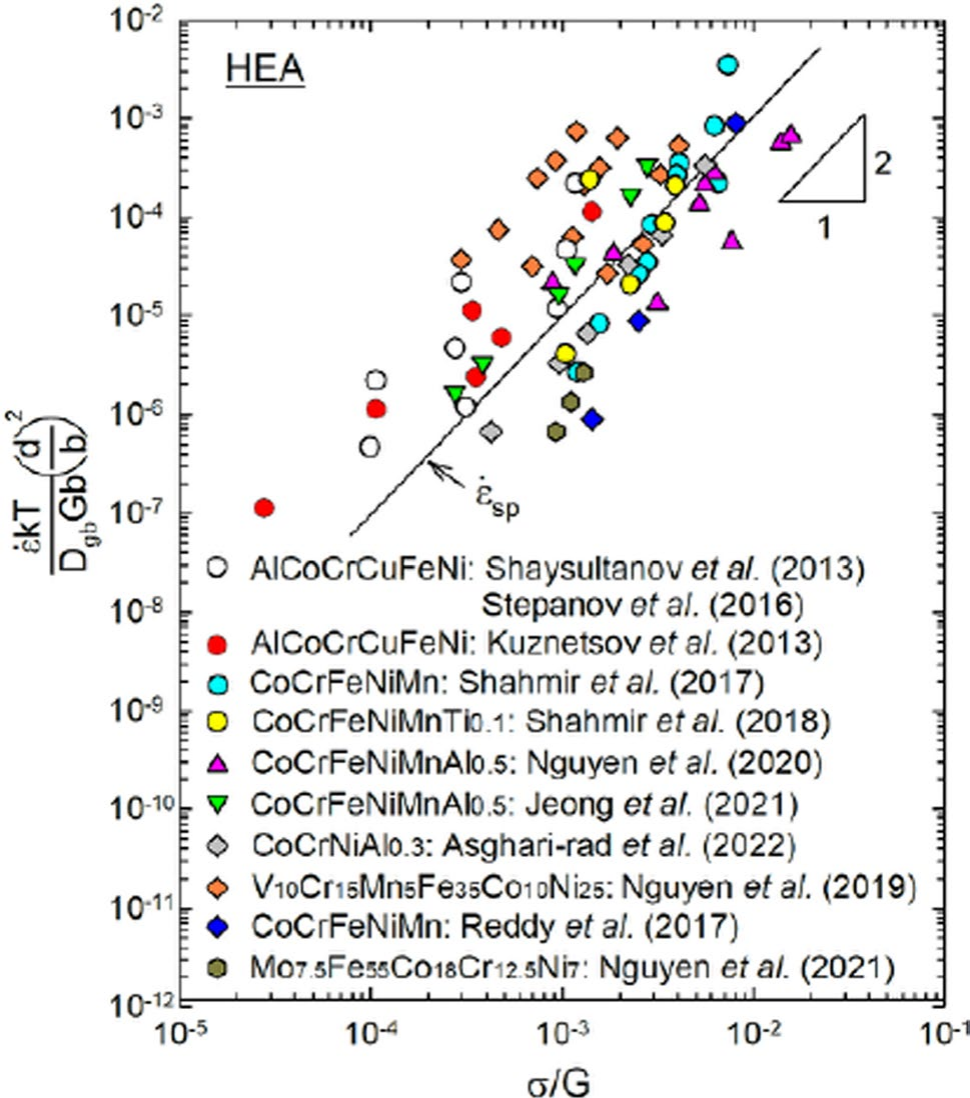
Temperature and grain size compensated creep rates plotted against normalized stress for high-entropy alloys [80–90] showing excellent agreement with the theoretical prediction for conventional superplasticity [55, 79].

A third example is shown in Fig. 14, where the grain size and temperature compensated creep rate is again plotted against the normalized stress and the solid line shows the theoretical prediction for grain boundary sliding in superplasticity estimated using Eq. (9) [55, 79]. The scattered datum points are taken from several reports describing the results obtained using high-entropy alloys (HEAs) [80–90]. Inspection shows that, although there are difficulties in determining the individual values of *D*_gb_ for these materials, all of the experimental points scatter around the predicted behaviour for ${\dot{\varepsilon }}_{\text{sp}}$. This demonstrates that the approach developed in this report is valid for a very wide range of material structures and this will include, for example, the very widely studied CoCrFeNiMn multicomponent HEA [91].

## Recent examples demonstrating the importance of creep in modern analyses

This report provides a summary of much of the research that has been developed to explain and interpret the flow behaviour of pure metals and metallic alloys when tested under creep conditions. No attempt was made in this review to examine additional associated topics such as the roles of threshold stresses, the effect of the stacking fault energy or the significance of precipitation. On the contrary, the report is designed instead to honour a former colleague, Professor Michael E. Kassner, and in this respect the review has concentrated on those topics that were of special interest to Kassner as documented in his major creep publications [92, 93]. It is important to note also that the flow mechanisms in creep were developed primarily for metals but similar behaviours are attained in ceramics [42, 94] and in geological materials [95] and this makes it relatively easy to construct deformation mechanism maps for materials such as olivine which is the most abundant mineral in the upper mantle of the Earth.

Since studies of creep may be traced back for slightly more than 100 years, it may be reasonable to anticipate that there is now a sufficient understanding of creep, with all of the flow mechanisms expressed by valid rate equations, that it is not necessary to conduct any additional creep experiments. In fact, this conclusion is not valid because the occurrence of creep plays a major role in many engineering operations and detailed evaluations are consistently needed to fully anticipate and predict the nature of creep deformation that will appear over a period of time in these structures. It is appropriate, therefore, to now give, in this concluding section, two very recent examples where creep played an important and hitherto unrecognized role. These examples are taken from the fields of civil or structural engineering and glaciology, respectively.

First, in structural engineering the radio telescope of Arecibo was built in the karst area of western Puerto Rico, where a sinkhole was available to accommodate a large collecting dish having a diameter of 305 m and a radius of curvature of 265 m and with a cable-mounted steerable radio receiver mounted at 150 m above the dish. This facility was completed in 1963, funded primarily by the U.S. National Science Foundation at a cost of over 9 million U.S. dollars, and it remained the largest single aperture telescope in the world for more than 50 years until surpassed by a telescope in China. It is important to note that the cables supporting the telescope had zinc-filled sockets at both ends, where the cables were inserted into cavities and then the individual wires were spread out before filling the cavities with molten zinc. In the initial specifications it was assumed, incorrectly, that this cable socket assembly would be stronger than the cable but, as will be demonstrated, these zinc-filled sockets were the primary factor relating to the failure of the telescope.

Earthquake damage led to the breakage of two supporting cables of the radio receiver in August and November 2020 and this led to questions concerning the strength of the remaining cables which were now required to fully support the suspended structure. In December 2020 the wires in the supporting cables started to break until finally the cables snapped so that the supported structure fell into the large dish and the telescope was destroyed.

The reason for this failure was not understood at the time but a committee was established by the Academies of Science, Engineering and Medicine to examine the reason for failure and their comprehensive report was published in 2024 [96]. This report contains a remarkably lengthy and comprehensive description of creep flow processes which follows much of the same approach as used in the earlier parts of this review. The overall conclusions from a very detailed analysis are best summarized by the following two quotations from the report:

Based on the observed Arecibo Telescope socket slip and the results of laboratory testing, it appears that all cable pullouts greater than ½ inch may involve zinc creep.

And later the following comment:

Plotting the stress/shear modulus estimate and temperature ratio for pure zinc on the reported zinc deformation map for 0.1 mm grains demonstrated that the Arecibo Telescope cable socket service lies in the regime of power-law creep (PLC).

In addition, a significant effect of temperature was noted in the following quotation:

Creep susceptibility depends strongly on the operating temperature relative to the material’s absolute melting temperature. Since zinc has a low melting temperature, which is required to prevent the molten socket material from heat-treating the steel wires during socket filling, it is potentially susceptible to creep at tropical temperatures.

All of these comments serve to emphasize the overwhelming importance of deformation by creep flow, as especially exacerbated by the high ambient temperatures, leading to damage that was not predicted simply because no comprehensive initial analyses were undertaken but which thereby led to the catastrophic failure by creep of a multi-million dollar high-level scientific facility. The absence of an extensive creep evaluation during the early lifetime of this facility is especially surprising when it is noted that a compilation of many creep results and a deformation mechanism map for pure zinc became readily available in 1982 [97].

For glaciology, it is important to first note that ice has an hexagonal close-packed (h.c.p.) crystal structure and therefore it is reasonable to anticipate the creep properties will have similarities to h.c.p. metals. In addition, the creep flow of ice is generally examined at temperatures that are very high relative to the melting temperature of ice and therefore at a high homologous temperature. The creep behaviour of ice was examined in an extensive review [98] that dated back to the early experiments conducted in the 1950s by Glen [99, 100] showing that the laboratory flow of ice lay within the regime of power-law creep with a stress exponent, *n*, in the range of ~ 3 to 4. In another review [101] there was a direct comparison between the creep data available for ice and the published experimental results for the three h.c.p. metals of Cd, Mg and Zn as well as a comparison between the creep of ice in the laboratory and measurements of creep in the field for glaciers [102] and ice shelves [103]. All of these results confirm the many similarities between the creep deformation of ice and conventional polycrystalline metals.

Generally, when examining creep processes associated with ice structures in the field, it is a standard practice to consider that the flow occurs as documented in the early experiments of Glen [99, 100] with conventional power-law creep. However, very recent research was developed to address specifically the problem of a potential rise in the sea level due to climate warming [104], where this is dependent upon the rate at which the ice sheets in the Antarctic and Arctic discharge ice into the sea. Since that rate is dependent upon the flow properties of ice, it seems reasonable to assume that the process will follow conventional data with a stress exponent of $n \approx {3}{-}{4}$. Nevertheless, careful analysis showed there was a temperature regime very close to the melting temperature where, due to the inherent pressure melting effect, there was a coexistence of liquid water at the crystal boundaries and this produced a linear-viscous flow with a stress exponent of $n \approx 1.0$. It was proposed that this effect was a direct consequence of diffusive pressure melting and refreezing at the grain boundaries and this new flow process would assist in stabilizing the ice sheets.

Thus, these experiments revealed a new and undocumented creep process having similarities to conventional diffusional creep with the deformation rate controlled by diffusion if there was a sufficient quantity of liquid water within the ice. As stated explicitly by the authors [104], their result *contradicts the prevailing thought that linear-viscous diffusion creep plays little role in glacier ice deformation* [105]. These data effectively demonstrate, therefore, that there remains an ability to conduct new and careful experiments and find flow processes that are not effectively described in the current conventional understanding of high-temperature creep. As noted in the discussion of these experiments, an interpretation of the creep data in glaciology is made complicated because there is a lack of sufficient information on the local grain size range and the precise water content, especially when it is noted that these parameters probably vary with depth within the glacier. Thus, the results will have important implications in future research on the flow and deformation of ice streams [106, 107] and Antarctic ice shelves [108].

## Summary and conclusions

1. Creep refers to the nonrecoverable plastic strain which accumulates with time when a crystalline material is subjected to an applied stress which is below the fracture stress. The subject of creep has attracted attention for more than 100 years so that there is now a good understanding of the principle flow mechanisms.
2. Equations are available describing the steady-state strain rates associated with the fundamental creep mechanisms. These cover diffusional creep and Harper-Dorn creep at low stresses and dislocation climb and glide at higher stresses. Equations are also available describing grain boundary sliding when the grain size is larger than the equilibrium subgrain size in conventional creep and grain boundary sliding when the grain size is smaller than the subgrain size in superplastic flow.
3. The processing of materials through the application of severe plastic deformation provides an opportunity for producing materials with grain sizes in the submicrometer or even the nanometer range. These small grain sizes are especially beneficial in producing materials that exhibit extensive superplasticity.
4. Direct comparisons are possible between the theoretical predictions and experimental data by plotting deformation mechanism maps or by directly comparing the predicted and experimental strain rates. Generally, there is very good agreement between theory and experiment.
5. Despite the long history of creep experiments, opportunities remain available for making significant contributions to the understanding of creep behaviour, for finding and developing new and significant creep mechanisms and for making early evaluations of the role of creep in future industrial operations. Two examples of opportunities in the field of creep are presented in the areas of structural engineering and glaciology.

## Data Availability

The data supporting the discussion in this study are fully available in published reports.
